# The association between low pH value and unfavorable neurological outcome among the out-of-hospital cardiac arrest patient treated by extra-corporeal CPR: sensitivity analysis

**DOI:** 10.1186/s40560-020-00470-3

**Published:** 2020-07-22

**Authors:** Yohei Okada, Takeyuki Kiguchi, Tetsuhisa Kitamura, Taku Iwami

**Affiliations:** 1 Department of Preventive Services, School of Public Health, Kyoto University, Kyoto, Japan; 2 Department of Primary Care and Emergency Medicine, Graduate School of Medicine, Kyoto University, Kyoto, Japan; 3 Kyoto University Health Service, Yoshida Honmachi, Sakyo, Kyoto, 606-8501 Japan; 4 Critical Care and Trauma Center, Osaka General Medical Center, Osaka, Japan; 5 Division of Environmental Medicine and Population Sciences, Department of Social and Environmental Medicine, Graduate School of Medicine, Osaka University, Osaka, Japan

## Abstract

This is the response to the comment from Dr. Romain Jouffroy and his colleague, on the manuscript “Association between low pH and unfavorable neurological outcome among out-of-hospital cardiac arrest patients treated by extracorporeal CPR: a prospective observational cohort study in Japan”. We performed sensitivity analysis based on the comment from them. It indicated that the results of primary analysis were robust even in considering their criticism.

To the editor,

We would like to thank Dr. Romain Jouffroy and his team for their interest and giving some suggestions for our research to investigate the association between pH value and neurological outcome among the out-of-hospital cardiac arrest (OHCA) patients treated by extracorporeal cardiopulmonary resuscitation (ECPR) [1, 2].

First, Dr. Romain and his team suggested that the time duration from OHCA occurrence to the blood test or no flow duration should be in the analysis as a covariate. Basically, we believe that it is unnecessary to adjust the low or no flow time duration to estimate the association between the pH value and outcome, because we think pH value is the representative of the duration and the quality of the resuscitation as described in the original article [1], and we assumed that the duration was not a confounder but a “parent” of the association between pH value and outcome in the causal inference. Further, they also suggested that categorization of the pH value to three groups could not reflect the pathophysiology accurately, and it should be treated as a continuous variable. In the primary analysis of the original article, the reason why we categorized the pH values was that it may be easy to interpret the association. However, we agree with the limitation that the categorization may not be able to represent the pathophysiology in more detail.

According to their suggestion, we performed the sensitivity analysis in which the time duration from call to blood test was added as a covariate, and the pH value was treated as continuous variable in the logistic model and indicated the association between the initial pH value and neurological outcome using the restricted cubic spline curve (Fig. 1). The detail of the method was described in additional file 1. This figure showed that the adjusted odds ratio for favorable neurological outcome was constantly low in the area of pH value less than 7.0, and it gradually increased in higher than 7.0. This result was almost the same as our primary analysis. Therefore, we believe that the result in primary analysis would be robust even though their concerns were taken into account.

**Fig. 1:**
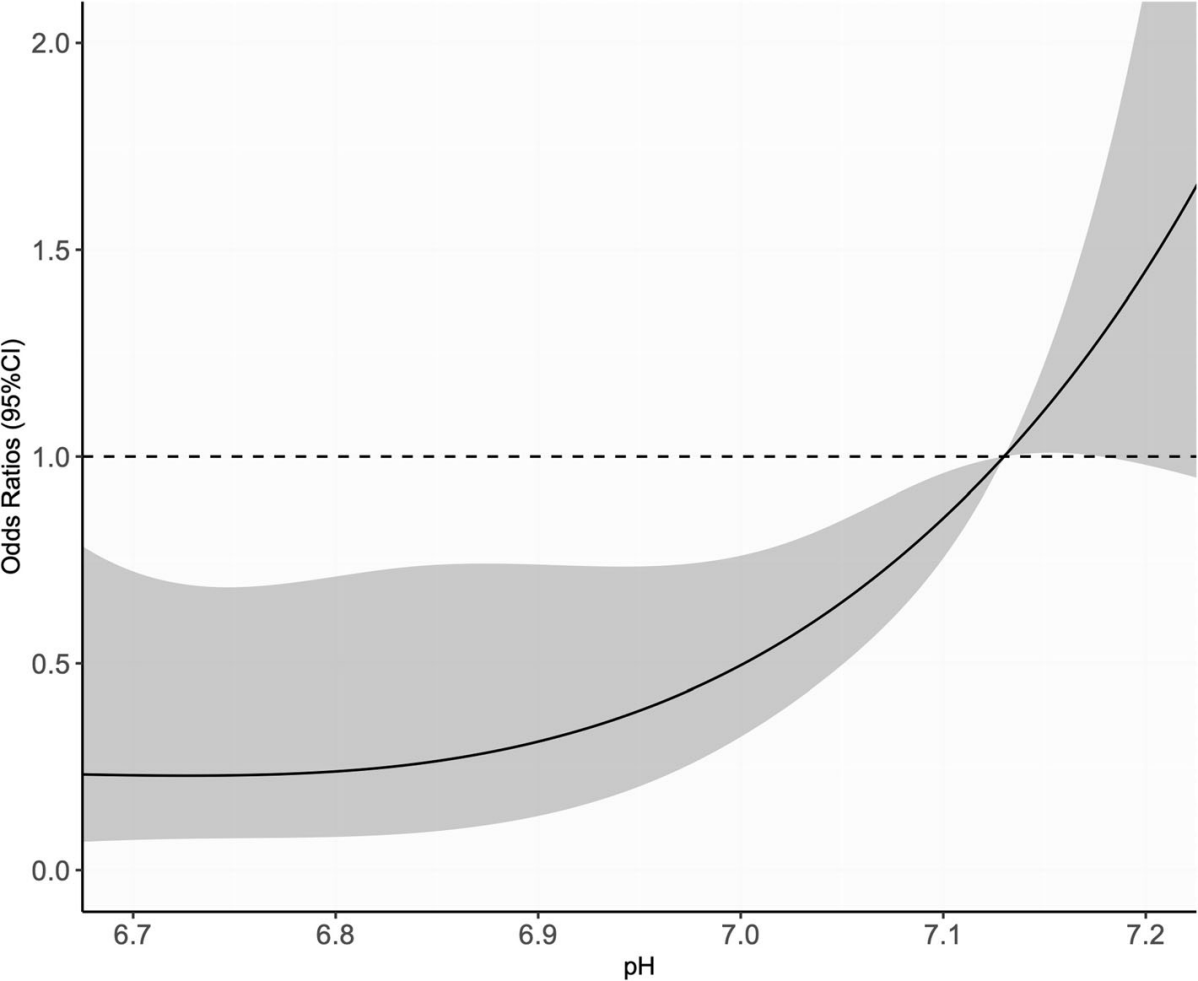
The results of sensitivity analysis. X-axis: Initial pH value before implementation of the extracorporeal cardio-pulmonary resuscitation (ECPR). Y-axis: The adjusted odds ratio and 95% confidence interval (CI) for favorable neurological outcome referred to median value in the pH ≥ 7.03 group (pH 7.13) were adjusted by sex (men, women), age (< 65, 65–74 and ≥ 75 years), witnessed by bystander, CPR by bystander, pre-hospital initial cardiac rhythm (shockable, non-shockable), cardiac rhythm on hospital arrival (shockable, non-shockable and ROSC) and the time from call to blood test. The method in detail was described in additional file 1

Second, we understood their criticism that the residual confounding in in-hospital phase might have exist. However, even if such an unmeasured in-hospital confounding would have existed, we believe that the association in our results would be robust. It is because the association was so obvious, and in order to change the result, it would be required to assume enough strong association between the potential unmeasured confounders and outcomes, based on the recent idea for unmeasured confoundin g[3, 4]; however, association between in-hospital factors(e.g., tracheal intubation or drug administration) and outcomes are generally small compared to pre-hospital factors [5, 6].

Third, we agree with the concern that the blood test results including pH value might influence on the decision-making of the implementation of extracorporeal cardio-pulmonary resuscitation, and it could be a selection bias as mentioned in limitation part [1]. However, this potential selection would not change the results of this study. Because we assumed that most cases influenced by the selection were decided to withdraw the ECPR due to low pH value, these patients would tend to die in the emergency department regardless of introduction of the ECPR. Therefore, we believe that it could not change the association of low pH value with unfavorable outcome.

Fourth, we understand their argument that the prevalence of return of spontaneous circulation in the tertile 1–3 groups were different; however, it was adjusted by logistic regression model in the primary analysis. Thus, it may not be a problem to interpret the result.

In conclusion, the result of sensitivity analysis based on the suggestion from Dr. Romain Jouffroy [2] indicated that the results of primary analysis were robust even in considering their criticism. We really appreciate for their suggestion on our analysis and giving the important opportunity to show the result of sensitivity analysis. We hope this process helps better understanding for readers.

## Supplementary information

**Additional file 1.** Method.

## Data Availability

Not applicable
