# Supplementary material for: The association between low pH value and unfavorable neurological outcome among the out-of-hospital cardiac arrest patient treated by extra-corporeal CPR: sensitivity analysis
Source: J Intensive Care. 2020 Jul 22;8:53. doi: 10.1186/s40560-020-00470-3 (PMC7374849; doi:10.1186/s40560-020-00470-3)
Supplement: Supplementary file 1 — Additional file 1. Method. [file 40560_2020_470_MOESM1_ESM.docx]

**Supplementary file**

**Method**

We used the same dataset of the patients (N=260) included in the primary analysis in the original article.[1] We showed the possible nonlinear relationship between pH value before extra-corporeal cardio-pulmonary resuscitation and the estimated odds ratio of favourable neurological outcome by restricted cubic spline curve using 4 knots at prespecified locations according to the distribution of pH value (the 5, 25, 75, and 95% percentiles) in the multivariable logistic regression model adjusted by following confounders: sex, age (< 65, 65–74, and ≥ 75 years), witnessed by bystander, CPR by bystander, pre-hospital initial cardiac rhythm (shockable, non-shockable), cardiac rhythm on hospital arrival (shockable, non-shockable, and return of spontaneous circulation), and the time from call to blood test. We used the R software (version 1.1.456; R Studio Inc.) with the “rms” package.[2]

**Reference**

1. Okada Y, Kiguchi T, Irisawa T, Yoshiya K, Yamada T, Hayakawa K, Noguchi K, Nishimura T, Ishibe T, Yagi Y *et al*: **Association between low pH and unfavorable neurological outcome among out-of-hospital cardiac arrest patients treated by extracorporeal CPR: a prospective observational cohort study in Japan**. *Journal of Intensive Care* 2020, **8**(1):34.

2. Harrell Jr FE, Harrell Jr MFE, Hmisc D: **Package ‘rms’**. *Vanderbilt University* 2019, **229**.
